# Enteral Feeding Progression Trajectories in the First Two Postnatal Weeks Are Associated with Motor Neurodevelopment at 24 Months in Preterm Infants: A Multicenter Cohort Study

**DOI:** 10.3390/nu18203314

**Published:** 2026-10-09

**Authors:** Maria Di Chiara, Gianluca Terrin, Victoire Van Reet, Benedicte Van Grambezen, Lucia Dito, Daniela Regoli, Barbara Caravale, Anais De Groote, Fiammetta Piersigilli

**Affiliations:** 1 Department of Maternal-Infantile and Urological Sciences, Policlinico Umberto I, Sapienza University of Rome, Viale del Policlinico 155, 00161 Rome, Italy; maria.dichiara@uniroma1.it (M.D.C.); lucia.dito@yahoo.it (L.D.); dani.regoli@virgilio.it (D.R.); 2 Department of Neonatology, Saint-Luc Hospital, University of Louvain, Avenue Hippocrate 10, 1200 Brussels, Belgium; victoire.vanreet@uclouvain.be (V.V.R.); benedicte.vangrambezen@uclouvain.be (B.V.G.); anais.degroote@uclouvain.be (A.D.G.); fiammetta.piersigilli@saintluc.uclouvain.be (F.P.); 3 Department of Developmental and Social Psychology, Sapienza University of Rome, 00185 Rome, Italy; barbara.caravale@uniroma1.it

**Keywords:** very-low-birth-weight infant, enteral nutrition, parenteral nutrition, trajectory clustering, Bayley Scales of Infant Development, motor neurodevelopment, gut–brain axis, human milk, preterm infant

## Abstract

Background/Objectives: Preterm infants are at substantial risk of neurodevelopmental impairment. Current guidelines recommend gradual advancement of enteral feeds during the first postnatal weeks, but the rate that is optimal for the individual infant remains uncertain. We modeled the day-by-day balance between enteral and parenteral nutrition during the first two postnatal weeks as longitudinal trajectory phenotypes and examined their association with neurodevelopment at 24 months of corrected age. Methods: Retrospective multi-center observational cohort of preterm infants (gestational age < 32 completed weeks or birth weight < 1500 g) from two centers managed under closely aligned nutritional protocols. The daily enteral-to-total-energy ratio on postnatal days 0–7 and 14 was clustered by K-means (K = 3) into parenteral-dominant (C0), moderate-progression (C1) and rapid-progression (C2) phenotypes. The primary outcome was the Bayley-III Motor composite at 24 months; Cognitive and Language composites at 24 months and the Motor composite at 12–18 months were additional outcomes. Linear mixed-effects models were adjusted for gestational age, birth-weight z-score and sex, with a random intercept for center. Results: Among 260 infants, three trajectory phenotypes were identified. At 24 months, the moderate-progression cluster had a higher Motor composite than the parenteral-dominant cluster (adjusted mean difference + 4.96 points; 95% CI, +1.51 to +8.41; *p* = 0.005; *n* = 194). In exploratory analyses of the other two domains, the unadjusted differences favoring the moderate cluster (cognitive + 7.1 points, *p* = 0.022; language + 6.9 points, *p* = 0.009) were abolished by adjustment for maturity (cognitive + 0.17 points, *p* = 0.94; language + 1.80 points, *p* = 0.45), whereas the motor difference remained statistically significant; the confidence intervals for these two domains do not exclude clinically relevant differences. The rapid-progression cluster did not differ significantly from either the parenteral-dominant or the moderate cluster, yielding a non-monotonic pattern. The motor association remained statistically significant after adjustment for baseline illness severity and was attenuated only marginally by downstream morbidities or the proportion of human milk. Conclusions: A moderate-progression enteral trajectory during the first two postnatal weeks was associated with better motor neurodevelopment at 24 months, whereas rapid advancement did not differ from parenteral dominance, so that the association was confined to a graded progression rather than to either extreme. The moderate-progression association was homogeneous across gestational-age strata (cluster × gestational age interaction, *p* = 0.23). These real-world findings are compatible with previously divergent evidence within a single non-monotonic dose–response framework.

## 1. Introduction

Preterm infants, particularly the most immature and those of lowest birth weight, remain at substantial risk of long-term neurodevelopmental disability, with rates of moderate-to-severe impairment reaching 10–25% in school-age follow-up cohorts [1]. Postnatal nutrition during the first weeks of life is one of the few modifiable determinants of this trajectory [2], yet, although guidelines recommend gradual advancement, the feeding strategy most favorable to neurodevelopment, and in particular the rate of advancement best suited to the individual infant, remains uncertain.

Parenteral nutrition is indispensable in the first postnatal days, when enteral intake alone cannot meet requirements, and an early amino acid supply sufficient to prevent catabolism and cumulative protein deficit remains an essential component of care. The historical paradigm of aggressive early parenteral nutrition (PN) has been challenged by recent evidence [3]. The randomized ProVIDe trial showed that additional early parenteral amino acids in extremely-low-birth-weight infants did not improve survival without neurodisability at 24 months, with a concerning signal of greater moderate-to-severe neurodisability in the intervention arm, and a subsequent systematic review confirmed that high protein intake may reduce survival without neurodisability and increase cognitive impairment at toddler age [4,5]. Consistently, PN-related neonatal hyperglycemia has been associated with motor and cognitive delay on Bayley-III at 24 months, and, in a cohort study, a more energy-aggressive parenteral-nutrition strategy was likewise associated with a lower motor score at 24 months [6,7]. The current ESPGHAN position papers reflect this shift, recommending gradual rather than aggressive enteral advancement [8,9]. Prolonged dependence on parenteral nutrition, however, is more often a marker of greater illness severity than a cause of harm in itself.

Converging evidence supports a beneficial role of the enteral route in the neurodevelopment of preterm infants. Enteral energy intake during the first weeks of life correlates positively with cerebral growth, whereas parenteral energy correlates negatively [10]; a randomized cohort replicated this directional pattern on visual evoked potentials [11], and a systematic review concluded that an enteral advantage is suggested though evidence remains limited [12]; moreover, early enteral feeding improves tolerance of concurrent parenteral nutrition and reduces early metabolic complications [13]. Mechanistically, the first two postnatal weeks coincide with the critical window of intestinal barrier maturation, early microbial colonization and vagal-afferent programming that have been proposed to constitute the gut–brain axis of the preterm infant [14]; early nutritional inputs are thought to modulate the evolving intestine [15], reduced early gut diversity predicts feeding intolerance [16], and early enteral feeding restores serum neurofilament light chain toward normal levels [17].

What constitutes the optimal rate of enteral progression is a central yet unresolved question that the randomized design is poorly suited to answer. A trial must, by construction, allocate infants in advance to a predetermined advancement schedule, for example a fixed “fast” versus “slow” daily increment [18]. In that trial, moreover, infants were randomized only once clinicians were ready to advance beyond 30 mL/kg/day, so that the comparison concerned infants in whom enteral feeding had already been established. Such fixed rules cannot adapt to the individual infant’s evolving gastrointestinal tolerance, so the contrast is between two arbitrary protocols rather than between the progression phenotypes that occur at the bedside; and, because the achievable pace depends on each unit’s practices, trial estimates are difficult to reproduce across settings. Analyzing the day-by-day feeding actually prescribed in routine care takes the opposite, complementary approach: clinicians titrate enteral advancement to each infant’s real-time tolerance, which itself reflects illness severity, so the observed trajectories approximate the progression physiologically attainable in practice, and the heterogeneity among them indicates which real-world pattern is most favorable. Trajectory methods have so far been applied to enteral feeding as a contrast between a faster and a slower course. A two-pattern description orders the extremes but leaves the shape of the relationship between them unspecified, and in particular cannot establish whether progression may also be too rapid. Addressing that question requires more than two phenotypes, and an observation window confined to the interval in which parenteral nutrition still supplies a substantial share of total energy.

To exploit this real-world signal while limiting single-unit idiosyncrasy, we designed a multi-center study in two tertiary neonatal intensive care units sharing harmonized data collection and closely aligned protocols for delivery-room stabilization, respiratory distress syndrome management and parenteral/enteral nutrition. We aimed, first, to characterize the trajectory phenotypes of the enteral-to-total-energy ratio over the first two postnatal weeks and, second, to examine their association with neurodevelopment, assessed by the Bayley-III composite scores at 24 months of corrected age (the Motor composite as the primary outcome, with the Cognitive and Language composites as additional domains) and by the Motor composite at 12–18 months, in preterm infants [19]. The design therefore allows the shape of the association to be examined rather than assumed, including whether the most rapid progression confers any advantage over a moderate one.

## 2. Materials and Methods

### 2.1. Study Design, Setting and Participants

This was a retrospective, multi-center, observational cohort study conducted in the level-III NICUs of Saint-Luc Hospital, University of Louvain (Brussels, Belgium) and Sapienza University of Rome–Policlinico Umberto I (Rome, Italy), covering consecutive admissions between January 2015 and December 2021 at the Rome unit and between January 2021 and December 2022 at the Brussels unit. Anonymized clinical data were retrieved from the institutional electronic health records and unit-specific nutritional databases, harmonized centrally according to pre-agreed definitions and merged into a single analytic file; no protected health information was transferred between institutions. The study is reported in accordance with the STROBE statement [20].

**Figure 1:**
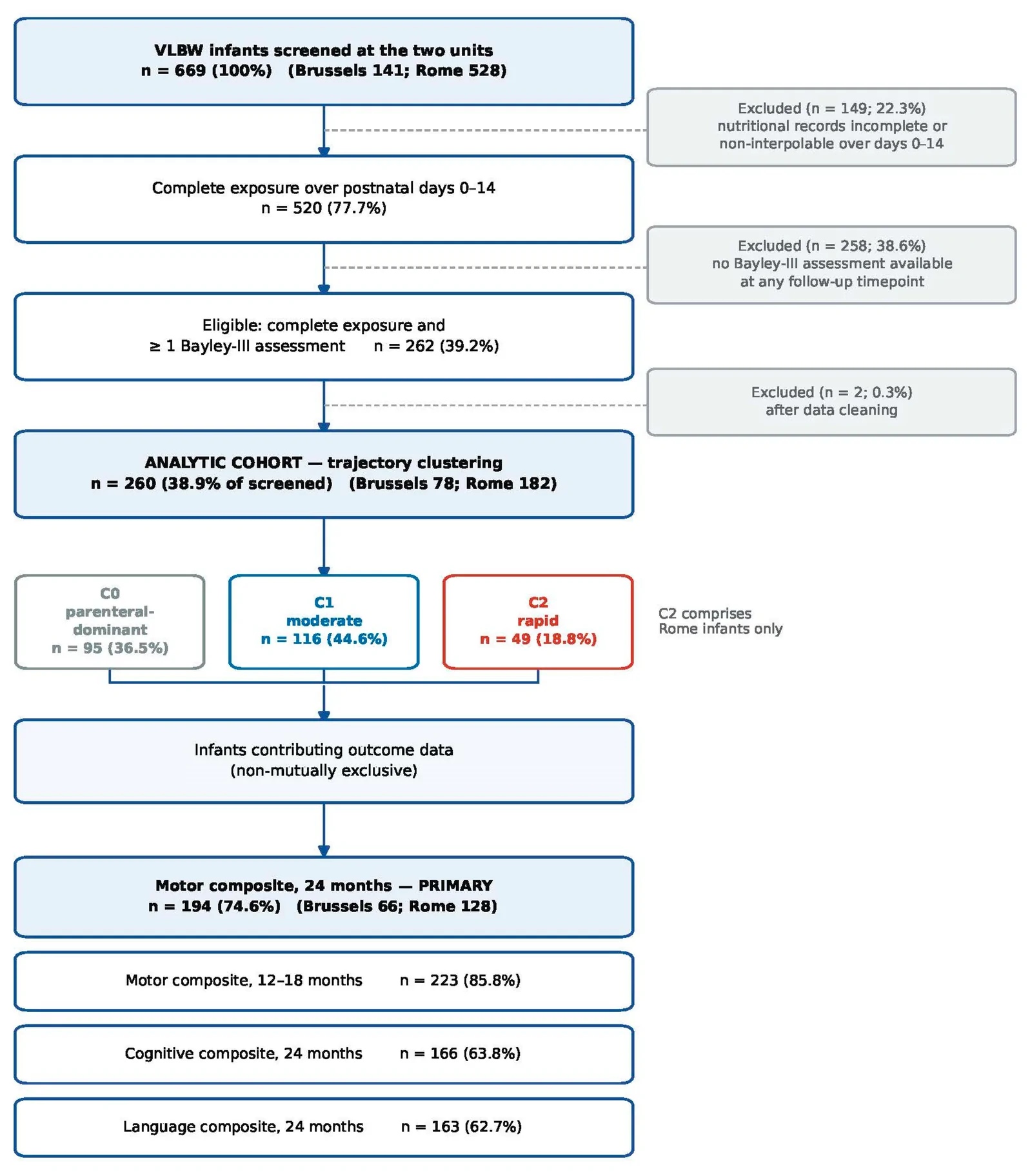
Participant flow. Percentages in the selection pathway refer to the 669 screened infants; percentages for the trajectory clusters and for outcome availability refer to the 260 infants of the analytic cohort. The predominant reason for non-inclusion was the absence of any developmental follow-up—258 infants had complete nutritional records but no Bayley-III assessment—rather than incomplete nutritional data, which accounted for 149 infants. Included and non-included infants did not differ in gestational age, birth weight, birth-weight z-score or sex (all *p* > 0.40; Table S7).

Eligible participants were live-born neonates meeting either of the pre-specified eligibility criteria (gestational age < 32 completed weeks or birth weight < 1500 g), admitted within the first 24 h of life. Pre-specified exclusions were death within the first 72 h of life, major congenital or chromosomal anomalies, inborn errors of metabolism, clinically apparent congenital TORCH infection, surgical gastrointestinal pathology requiring fasting beyond day 14, and transfer to or from another tertiary unit during the first 14 days. Infants with incomplete, non-interpolable nutritional records during the exposure window were excluded (Figure 1).

### 2.2. Nutritional Protocol

Both units managed parenteral and enteral nutrition under closely aligned protocols based on the ESPGHAN recommendations, whose general framework has been described in previous reports from the participating investigators [8,9,21]; the protocol reported here is the one in force at the two units during the study period, with ranges reflecting birth-weight-dependent and tolerance-dependent modulation. Parenteral nutrition was started within the first 24 h of life through central venous or peripherally inserted central catheter access; the total daily fluid intake (combined parenteral and enteral) was commenced at approximately 70–90 mL/kg/day and gradually increased, by about 10–20 mL/kg/day, to a target of approximately 150–180 mL/kg/day. Parenteral nutrition was prescribed individually each day on the basis of clinical condition, laboratory results and body weight, with macronutrient targets following the ESPGHAN recommendations [22] and was continued until full enteral feeding was achieved. Starting doses, target doses and the time allowed to reach the target for energy, amino acids, dextrose and lipid emulsion, stratified by birth weight, are reported in Supplementary Table S5. Enteral nutrition was initiated within the first 96 h of life, using mother’s own milk given fresh whenever available, as minimal enteral feeding of 10–20 mL/kg/day divided into four to eight feeds; the daily enteral volume was then advanced by 15–30 mL/kg/day according to birth weight and tolerance, with fortification of human milk started once the enteral volume reached 80 to 100 mL/kg/day according to birth weight at the Rome unit (FM 85, Nestlé, Vevey, Switzerland) and 80 mL/kg/day at the Brussels unit ((Pre NAN HMF, Nestlé)). Donor human milk was not available at either unit. Probiotics were administered routinely at the Brussels unit and were not administered at the Rome unit. Preterm formula was given when mother’s own milk was unavailable or insufficient. In the presence of feeding intolerance (significant abdominal distension, emesis, ileus with visible intestinal loops, blood in the stools, or a gastric residual that was bloody or bilious or exceeded 50% of the preceding feed) or hemodynamic instability, enteral feeding was withheld for at least 6 h and re-evaluated before resumption. Gastric residuals were not measured routinely. Full enteral nutrition was defined as 120 kcal/kg/day. Throughout the admission, infants received developmental care, with minimal handling and protection from light, noise and painful procedures [23].

### 2.3. Exposure Assessment and Trajectory Clustering

For each infant and each of postnatal days 0–7 and 14, the enteral-to-total-energy ratio (enteral fraction = EN/(EN + PN)) was computed. This ratio was preferred to enteral volume, to the percentage of enteral feeds and to days to full feeds because energy, rather than volume, is the quantity in which nutritional targets are expressed and in which the two routes are interchangeable: volume-based measures ignore the differing energy densities of milk and of the parenteral solution, whereas days to full feeds reduces the entire course to a single threshold-dependent value that is undefined when full feeds are never reached and discards the shape of the progression. The observation window through postnatal day 14 was pre-specified on biological grounds as the hypothesized critical period of gut–brain maturation [14]. Clinically, the same interval is the phase of greatest instability, during which parenteral nutrition still contributes a substantial share of total energy and the balance between the two routes is adjusted daily at the bedside. Within that window, intakes were sampled on days 0 to 7 and on day 14, these being the timepoints recorded in both source databases. Brief gaps (≤2 consecutive days) were filled by linear interpolation. Heterogeneity in the trajectory was modeled by K-means clustering of the nine-dimensional vector [19]; K = 3 was pre-specified (parsimony, clinical interpretability, internal validity indices for K = 2–6), with Euclidean distance, k-means++ initialization, 30 restarts and a fixed seed (42), implemented in scikit-learn; the nine variables are bounded on a common 0–1 scale and were therefore not standardized. Centroids were ordered by mean enteral fraction at day 7 and labeled C0 (parenteral-dominant), C1 (moderate-progression) and C2 (rapid-progression). The investigators who performed the analysis were blinded to the neurodevelopmental outcomes during cluster derivation. The clusters are empirical phenotypes rather than prescribed advancement schedules; to make them clinically interpretable, the mean enteral fraction at postnatal day 7 was 10% in C0, 51% in C1 and 93% in C2 (corresponding to mean enteral intakes of 12, 53 and 90 kcal/kg/day against parenteral intakes of 94, 57 and 5 kcal/kg/day), and at day 14 it was 34%, 97% and 98%, respectively. Day 7 therefore distinguishes all three phenotypes, whereas day 14 identifies those that had not progressed. Interpolation was required for 176 of the 2340 exposure cells (7.5%), of which 76% were day-0 values; 105 of the 260 infants had all nine timepoints directly observed.

### 2.4. Outcomes and Covariates

Outcomes were the Bayley-III composite scores at 24 months of corrected age, assessed by trained psychologists or pediatricians blinded to exposure and to the neonatal nutritional history, using the language-adapted test (French in Brussels, Italian in Rome); the Motor composite, the primary outcome, is based on observed performance rather than verbal responses and is therefore unaffected by the language of administration, unlike the Language composite. The primary outcome was the Motor composite at 24 months; the Cognitive and Language composites at 24 months and the Motor composite at 12–18 months were additional outcomes, and motor delay (Motor composite < 85) at 24 months was a dichotomous outcome. Confounders were selected a priori from a directed acyclic graph: gestational age, birth-weight z-score and sex. Early morbidities (mechanical ventilation, bronchopulmonary dysplasia, necrotizing enterocolitis, intraventricular hemorrhage, late-onset sepsis) were excluded from the primary model as downstream mediators [24,25].

### 2.5. Statistical Analysis

Continuous composites were analyzed with linear mixed-effects regression (cluster + gestational age + birth-weight z-score + sex; random intercept for center; REML); the three pairwise contrasts were obtained by re-parameterizing the reference. The dichotomous outcome used logistic regression with center as a fixed effect. Effect measures are reported with 95% CIs; no a priori or post hoc power calculation was performed [26,27]. The minimum difference detectable with 80% power in the primary contrast was 4.9 points. The adjustment strategy comprised three tiers: Tier A (primary confounders); Tier B (baseline-severity sensitivity, adding 5 min Apgar, surfactant and antenatal corticosteroids); and Tier C (controlled-direct-effect analyses adding, one at a time, mechanical ventilation, bronchopulmonary dysplasia, necrotizing enterocolitis, intraventricular hemorrhage, late-onset sepsis, postnatal corticosteroids or the proportion of human milk). Throughout this report, pre-specified denotes choices that were fixed before the outcome models were fitted, and analyses developed subsequently are identified as post hoc. The primary confounder set, the exclusion of mechanical ventilation, and the three sensitivity analyses (exposure windows 0–7 and 0–28 days, center-stratified estimation, complete-case) were pre-specified; Tier B/C (other than mechanical ventilation) and the analyses of the Cognitive and Language domains were post hoc. The Bayley-III Motor composite at 24 months was the single primary outcome, pre-specified on biological and empirical grounds (the hypothesized critical window of gut–brain maturation; the domain-specific motor signal reported in the SIFT trial); no multiplicity adjustment was therefore applied to the primary contrast. The Cognitive and Language composites at 24 months were analyzed as exploratory outcomes and are reported without confirmatory intent: their smaller samples limit precision, and the Language composite was administered in two different languages across centers, which precludes a pooled inferential interpretation of that domain. Analyses used Python 3.10.12 (statsmodels 0.14.6, scikit-learn 1.7.2, pandas 2.3.3), seed = 42. Baseline characteristics and unadjusted composite scores were compared across clusters using the Kruskal–Wallis test for continuous variables (with the Mann–Whitney U test for pairwise comparisons) and Fisher’s exact or chi-square test for categorical variables, without correction for multiplicity. Because the trajectory clusters differed in gestational age, we additionally examined whether the cluster–outcome association was modified by maturity. A cluster × gestational-age interaction was tested by likelihood ratio, with gestational age entered as a continuous variable and, in a supporting model, as four strata (<28, 28–30, 30–32 and ≥32 completed weeks, the last comprising infants eligible by birth weight alone). Stratum-specific estimates were obtained by ordinary least squares with HC3 robust standard errors, adjusted for gestational age, birth-weight z-score, sex and center, and are reported as exploratory and descriptive; contrasts were declared non-estimable where a cluster contained fewer than five infants within a stratum, rather than estimated by model extrapolation. Heterogeneity of stratum-specific estimates was quantified by Cochran Q and I^2^. These analyses were post hoc. Sensitivity analyses restricted by eligibility criterion were performed post hoc, in response to peer review.

### 2.6. Ethics

This was a retrospective analysis of routinely collected anonymized clinical data extracted from the institutional electronic health records; no additional clinical procedures were performed. It was conducted in accordance with the Declaration of Helsinki (2013) and approved by the research ethics committees of both centers (Saint-Luc, Brussels: 2023/06FEV/061; Sapienza–Policlinico Umberto I, Rome: protocol no. 5089, 13 September 2018). Given the retrospective design and the exclusive use of anonymized data, the requirement for individual informed consent was waived by both committees.

## 3. Results

### 3.1. Participant Flow

Of the 669 preterm infants identified across the two units, 520 had nutritional records sufficiently complete over postnatal days 0–14; of these, 262 also had at least one Bayley-III assessment available, and two were excluded after data cleaning, leaving 260 infants for trajectory clustering (Brussels, 78; Rome, 182). The predominant reason for non-inclusion was therefore the absence of any developmental follow-up (258 infants had complete exposure records but no Bayley assessment) rather than incomplete nutritional data, which accounted for 149 infants. Infants in the analytic cohort did not differ from those not included with respect to gestational age (median 29 vs. 30 weeks; *p* = 0.64), birth weight (1235 vs. 1240 g; *p* = 0.56), birth-weight z-score (*p* = 0.56) or sex (50.4% vs. 53.8% male; *p* = 0.44); non-included infants were, however, disproportionately from the Rome site, which screened the larger number of admissions. The Bayley-III Motor composite at 24 months was available for 194 infants (Brussels, 66; Rome, 128), the Motor composite at 12–18 months for 223, the Cognitive composite at 24 months for 166, and the Language composite at 24 months for 163 (Figure 1; by center, Supplementary Figure S3). Because the two eligibility criteria were combined with an inclusive OR, 49 of the 260 infants in the analytic cohort (18.8%) were eligible on gestational age alone, with a birth weight of 1500 g or above (median 1655 g, range 1500 to 2370 g; gestational age 29 to 31 weeks); 37 (14.2%) were eligible on birth weight alone, at 32 weeks of gestation or more; and 174 (66.9%) met both criteria. Neonatal morbidities were similar in the two groups except for intraventricular hemorrhage of grade II or above, which was more frequent among infants not included (Supplementary Table S7).

### 3.2. Baseline Characteristics

**Figure 2:**
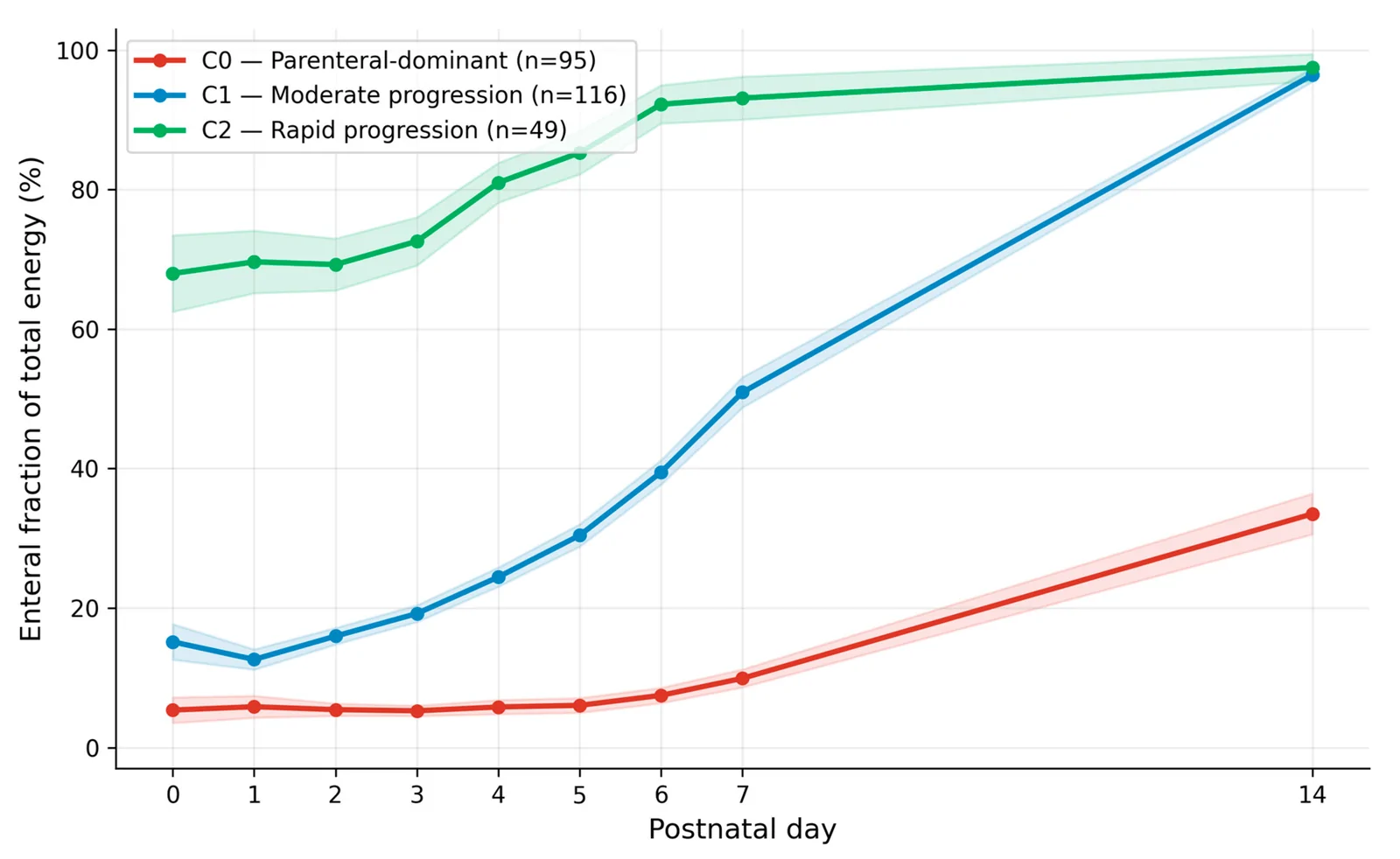
Mean enteral-to-total-energy ratio across postnatal days 0–14, by K-means trajectory cluster (C0 parenteral-dominant; C1 moderate-progression; C2 rapid-progression). Shaded bands denote the standard error of the mean. Curves are computed on the interpolated exposure matrix used for clustering (brief gaps of up to two consecutive days filled by linear interpolation, 176 of 2340 cells, 76% of them on day 0); means computed on directly observed values only differ appreciably at day 0 for the rapid-progression cluster (57% instead of 68%). The numbers of infants with a directly observed value were 127, 238, 255, 259, 257, 258, 257, 256 and 257 at days 0 to 7 and at day 14, respectively.

Baseline characteristics, overall and by cluster, are reported in Table 1. Median gestational age rose across the parenteral-dominant, moderate and rapid clusters (28, 30 and 31 weeks) as did the median birth-weight z-score (−0.6, +0.2, +0.6). The rapid-progression cluster was composed entirely of infants enrolled at the Rome site. Mean enteral-fraction trajectories by cluster are displayed in Figure 2. The three phenotypes were unevenly distributed between the two centers: the parenteral-dominant and moderate-progression clusters were represented at both sites, whereas all 49 infants in the rapid-progression cluster were from the Rome center (Table 1). The clusters differed significantly at baseline in gestational age, birth-weight z-score, head circumference at birth, 5 min Apgar score and center (all *p* < 0.001), whereas the remaining baseline characteristics did not differ. Standardized mean differences for each pairwise comparison are reported in Supplementary Table S6. Neonatal morbidities, which occur during or after the exposure window, are reported by cluster in Supplementary Table S8. Over postnatal days 0 to 14, 72 of the 253 infants with feeding data (28.5%) received mother’s own milk exclusively, 147 (58.1%) received both mother’s own milk and preterm formula and 34 (13.4%) received preterm formula exclusively; donor human milk was not available and no infant received it. The median proportion of mother’s own milk in the enteral volume was 0.57 in the parenteral-dominant cluster, 0.78 in the moderate cluster and 0.24 in the rapid cluster.

### 3.3. Primary Outcome

For the Bayley-III Motor composite at 24 months, the moderate-progression cluster scored higher than the parenteral-dominant cluster after adjustment for gestational age, birth-weight z-score, sex and center (adjusted mean difference, +4.96 points; 95% CI, +1.51 to +8.41; *p* = 0.005; standardized effect size, 0.33; 95% CI, 0.10 to 0.56). The rapid-progression cluster did not differ significantly from the parenteral-dominant cluster (+1.36 points; 95% CI, −3.42 to +6.15; *p* = 0.58) nor from the moderate cluster (−3.59 points; 95% CI, −7.93 to +0.74; *p* = 0.10). Unadjusted scores by cluster are presented in Table 2. The distribution of individual Motor composite scores by trajectory cluster is shown in Supplementary Figure S1.

**Figure 3:**
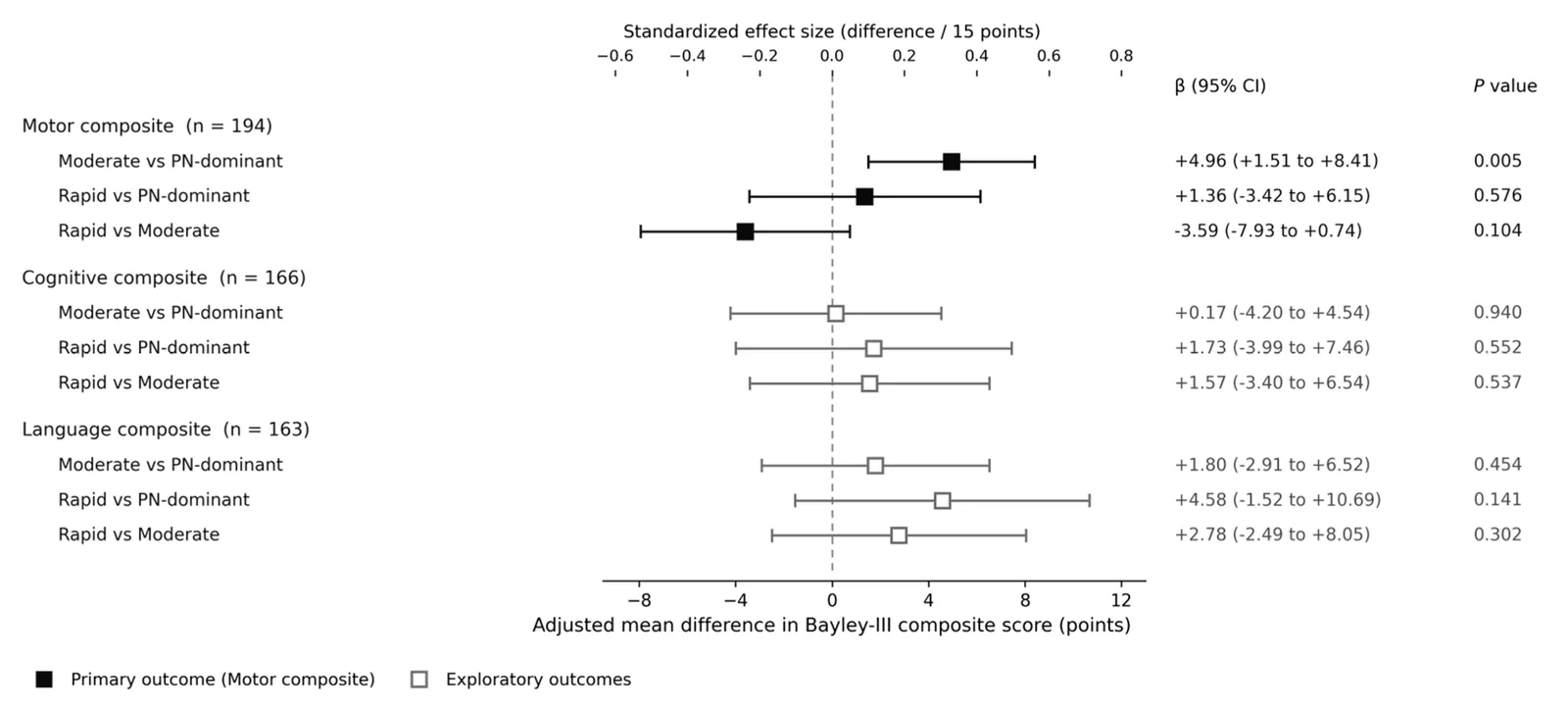
Adjusted associations (linear mixed-effects model: cluster + gestational age + birth-weight z-score + sex; random intercept for center) between trajectory cluster and the Bayley-III Motor, Cognitive and Language composites at 24 months, for all three pairwise contrasts. Squares denote the adjusted mean difference and horizontal lines the 95% CI; the dashed line indicates no difference. Filled squares denote the primary outcome and open squares the exploratory outcomes. The upper axis expresses the same differences as standardized effect sizes, obtained by dividing by the normative standard deviation of 15 points. PN = parenteral nutrition. The Motor composite is the single pre-specified primary outcome; the Cognitive and Language composites are exploratory, post hoc outcomes, and the Language composite was administered in two different languages across centers.

The adjusted between-cluster mean differences for the Bayley-III Motor, Cognitive and Language composites at 24 months, estimated from the primary linear mixed-effects model and presented for all three pairwise contrasts, are summarized in Figure 3.

### 3.4. Secondary and Additional Analyses

In exploratory analyses of the remaining two domains, the unadjusted scores favored the moderate cluster in both: cognitive 95.8 (SD 13.5) versus 88.7 (14.0) in the parenteral-dominant cluster (*p* = 0.022) and language 87.8 (12.5) versus 80.9 (14.7) (*p* = 0.009); both differences, however, were abolished by adjustment for gestational age, birth-weight z-score, sex and center (cognitive, moderate vs. parenteral-dominant +0.17 points; 95% CI, −4.20 to +4.54; *p* = 0.94; language, +1.80 points; 95% CI, −2.91 to +6.52; *p* = 0.45), in contrast with the motor difference, which remained statistically significant. These two domains were available in fewer infants (cognitive *n* = 166, language *n* = 163) and their confidence intervals do not exclude differences of a magnitude comparable to the motor estimate; they therefore neither support nor exclude an association and are reported as exploratory (Supplementary Table S1). The Language composite was administered in French at the Brussels unit and in Italian at the Rome unit, and the pooled estimate is therefore reported descriptively (rapid vs. parenteral-dominant, +4.58 points; 95% CI, −1.52 to +10.69; *p* = 0.14) (Figure 3). For the Motor composite at 12–18 months the moderate-versus-parenteral-dominant contrast was +4.49 points (95% CI, +0.53 to +8.45; *p* = 0.026). A dichotomous analysis of motor delay (<85) at 24 months is provided as Supplementary Table S2 (motor delay occurred in 29.0%, 4.3% and 19.4% of infants in the parenteral-dominant, moderate and rapid clusters respectively; moderate vs. parenteral-dominant, adjusted OR 0.25; 95% CI, 0.07 to 0.88; *p* = 0.031).

The primary motor contrast was not reduced by additional adjustment for baseline illness severity (5 min Apgar, surfactant and antenatal corticosteroids; +5.08 points; 95% CI, +1.59 to +8.57; *p* = 0.004) and changed by no more than 10% when downstream morbidities or the proportion of human milk were entered as controlled direct effects (Supplementary Table S3). It was non-significant in the 0–7-day window, maximal at 0–14 days and intermediate at 0–28 days; significant at the Brussels site and non-significant at the Rome site; and remained statistically significant in the complete-case analysis, which retained only the 81 infants whose nine exposure timepoints were all directly observed, with no interpolated value (+8.24 points; 95% CI, +2.20 to +14.27; *p* = 0.007); infants with and without interpolated values did not differ in gestational age, birth-weight z-score or motor outcome (all *p* > 0.15). No study-related adverse events were applicable given the retrospective design. The association between trajectory cluster and the Motor composite was not modified by gestational age: the cluster × gestational-age interaction was not significant with gestational age continuous (*p* = 0.23), across the four gestational-age strata (*p* = 0.68), or within the Rome center (*p* = 0.28). The moderate-versus-parenteral-dominant estimate was positive in all four strata (+5.66, +3.07, +3.39 and +2.60 points from the most to the least immature) with no detectable heterogeneity (Cochran Q *p* = 0.97; I^2^ = 0%), although within-stratum power for a 5-point difference was 18–47% and no stratum-specific estimate reached significance. The adjusted estimate was essentially invariant across maturity, from +5.5 points at 26 weeks to +4.8 points at 32 weeks, and was unchanged when gestational age was modeled with a quadratic term (+5.06; *p* = 0.004) and when infants eligible by birth weight alone were excluded (+5.10; *p* = 0.010). The rapid-progression cluster was absent below 28 weeks and comprised five infants between 28 and 30 weeks, so its stratum-specific contrasts remain descriptive (Supplementary Figure S2 and Table S4). The estimate was likewise unchanged when infants eligible by gestational age alone were excluded (+4.63 points; 95% CI, +0.88 to +8.38; *p* = 0.016) and when the analysis was restricted to infants meeting both eligibility criteria (+4.85 points; 95% CI, +0.54 to +9.15; *p* = 0.027). Because the rapid-progression cluster occurred only at the Rome site, contrasts involving that cluster are identified within the Rome center alone; within that center the moderate cluster scored 4.38 points above the rapid cluster (95% CI, −0.29 to +9.05; *p* = 0.065). Replacing the random intercept for center with center as a fixed effect, which restricts every comparison to infants managed at the same site, left the primary estimate essentially unchanged (+4.80 points; 95% CI, +1.35 to +8.26; *p* = 0.007). All sensitivity analyses of the primary contrast are summarized in Table 3.

## 4. Discussion

In this multi-center retrospective cohort of preterm infants, we identified three discrete enteral-progression phenotypes. After adjustment, infants on a moderate-progression trajectory had higher Bayley-III Motor composite scores at 24 months than infants on a parenteral-dominant trajectory; the motor association remained statistically significant after adjustment for maturity whereas the unadjusted differences in the cognitive and language domains did not, although the precision of those two exploratory analyses does not allow an association to be excluded in either; and the relationship across phenotypes was non-monotonic: rapid progression was not statistically distinguishable from parenteral dominance, so that both a prolonged parenteral-dominant course and an immediately rapid advancement were associated with lower motor scores than a moderate, graded progression.

The lower end of this dose–response is concordant with prior work: a slow-progression pattern has been associated with head-size growth faltering and neurodevelopmental impairment, a longer time to full enteral feeding predicts worse neurodevelopment without mediation by growth failure, and a Cochrane review supports earlier enteral feeding [28,29,30]. The upper end mirrors an apparently divergent strand: in the SIFT trial a faster feed increment did not improve survival without disability and was associated with an unexpected, domain-specific increase in motor impairment not explained by necrotizing enterocolitis or sepsis [18]; the STENA 2-year follow-up showed no benefit of rapid advancement [31]; and aggressive early parenteral nutrition trials found no benefit with signals of harm [4,5]. The two designs differ in a way that bears on this comparison: in the trials the rate of advancement was assigned, whereas here it was achieved, so that the rapid-progression cluster comprises infants who advanced quickly because they tolerated it. The absence of an advantage at the fast end therefore means something different in each, namely that imposing a faster increment confers no benefit, and that among infants able to sustain it a faster progression was not associated with a better motor outcome than a graded one; it is the agreement of these two independent routes that the interpretation proposed here rests upon. Our findings reconcile these strands within a single non-monotonic curve, though the direct rapid-versus-moderate contrast did not reach significance and is best described as a plateau.

The first of the three reports cited above is the closest in design to the present analysis and merits direct comparison. It clustered the absolute daily enteral volume over the first 56 postnatal days in a single-center cohort of extremely preterm infants born at 23 to 27 weeks, resolved two progression patterns, and analyzed a binary composite of neurodevelopmental impairment in which a cognitive or motor composite below 85 was pooled with moderate-to-severe cerebral palsy and profound sensory deficits. Three differences follow from those choices. The two patterns in that cohort became statistically distinguishable only from postnatal day 13 onward, so the comparison carries little information about the interval examined here, which closes at day 14. Absolute enteral volume and the enteral-to-total-energy ratio are distinct constructs: the former describes how quickly feeds are advanced, the latter the extent to which enteral intake has replaced parenteral support. And a binary composite outcome, by aggregating domains, does not permit an association to be attributed to any one of them. The two analyses are therefore complementary rather than overlapping: the earlier work characterized the lower end of the dose–response relationship in a more immature population, whereas the present cohort, by resolving an additional phenotype, extends the description to its upper end.

None of the biological pathways discussed in this paragraph were measured in the present cohort, so the account that follows is offered as a plausible mechanism rather than as an explanation established by the present data. The motor-domain specificity is biologically coherent and aligns with the SIFT motor signal. The first two postnatal weeks coincide with gut–brain maturation, the trajectory of early gut colonization predicts later Bayley scores, and early enteral feeding restores neurofilament light chain toward normal levels [14,17,32]. A moderate exposure may provide sufficient luminal stimulus without exceeding the immature gut’s capacity. Milk type may also contribute: rapid advancement was achieved with a lower proportion of human milk, the SIFT adverse signal was confined to formula-fed infants, and human-milk feeding is associated with better preterm neurodevelopment in a dose-dependent manner [18,33,34]. The association did not appear mediated by somatic growth, consistent with Yoon [29].

The three phenotypes differed substantially at birth in gestational age, birth-weight z-score and head circumference. This is expected rather than incidental: tolerance of enteral advancement is itself a function of maturity, so the more mature infants are those in whom a faster progression can be achieved. Two features of the analysis argue against the interpretation that the phenotypes are simply a restatement of maturity. First, gestational age and birth-weight z-score were included in every model, and the same adjustment that abolished the unadjusted differences in the cognitive and language domains left the motor difference essentially intact; had the clusters been a proxy for maturity, all three domains would have been attenuated alike. Second, the association did not vary with maturity: the cluster by gestational-age interaction was not significant, the estimate was positive in every gestational-age stratum with no detectable heterogeneity, and the adjusted estimate was almost constant from 26 to 32 weeks. Within-stratum precision was nevertheless limited and no stratum-specific estimate reached significance, so these analyses should be read as support for the robustness of the overall estimate rather than as evidence of an effect within each stratum. In an observational design maturity and feeding behavior cannot be fully separated, and residual confounding along this axis cannot be excluded.

The association was robust: it remained statistically significant after adjustment for baseline illness severity and was attenuated only marginally by downstream morbidities or human-milk fraction, and an E-value indicated that a moderately strong unmeasured confounder would be required to explain it away [25,35]. No minimal clinically important difference has been formally established for the Bayley-III composite scores; a difference of 5 points was adopted as the minimal clinically important difference in a randomized trial of supplemental donor human milk in very-low-birth-weight infants [36], and the adjusted motor difference observed here is of that magnitude. The contrast estimated on the Motor composite at 12–18 months was of similar direction and magnitude, and included 66 infants who did not contribute to the 24-month analysis, so that the two estimates are not simply the same infants measured twice. The earlier assessment is nonetheless corroborative rather than decisional: its predictive value for later function is lower than that of the assessment at 24 months, which remains the reference outcome. Clinically, these data point to gradual establishment of enteral feeding during the first two postnatal weeks as the modifiable target, consistent with the ESPGHAN recommendation for gradual advancement [9].

Strengths include the multi-center, real-world design with harmonized data collection, characterization of the exposure as a complete daily trajectory, a pre-specified exposure window, a priori confounder selection, and the observation that the motor association survived adjustment for maturity while the unadjusted differences in the other two domains did not [24]. Limitations follow from the retrospective design: the rapid cluster was composed entirely of Rome infants, so that trajectory and center cannot be separated for this cluster and the random intercept for center cannot remedy this; its contrasts are therefore descriptive, and any numerically higher language score in this cluster would in any case be confounded by the language of test administration; the motor association reached significance at Brussels but not at Rome, indicating between-center heterogeneity; the cognitive and language domains were available in fewer infants (*n* = 166 and *n* = 163) and were analyzed as exploratory outcomes whose confidence intervals do not exclude clinically relevant differences, so that domain specificity cannot be claimed; the dichotomous analysis was underpowered; and follow-up was limited to 24 months. The cognitive/language and Tier B/C analyses were post hoc and exploratory. Two further limitations follow from the structure of the cohort. First, 260 of the 669 screened infants entered the analytic cohort and 194 contributed the primary outcome, predominantly because developmental follow-up was unavailable; although included and non-included infants were comparable in gestational age, birth weight and sex, selection cannot be formally excluded, and infants who died after postnatal day 7 contribute to the characterization of the exposure but not to the outcome models. Second, stratification by gestational age, while showing no effect modification, concentrates rather than removes confounding by center: Brussels contributed almost exclusively moderate-progression infants above 30 weeks and Rome almost exclusively parenteral-dominant infants below 28 weeks, so that fully within-center stratified estimates were largely non-estimable. The stratified analyses should therefore be read as exploratory support for the robustness of the primary estimate rather than as subgroup estimates. The observation window was deliberately restricted to the first two postnatal weeks. This is the period of greatest clinical instability, when parenteral nutrition still supplies a substantial share of total energy and the balance between the two routes is titrated day by day at the bedside; it is therefore the interval in which early nutritional management is both most consequential and most modifiable. The corresponding limitation is that these data describe the establishment of enteral feeding and not its later course, and cannot be extrapolated to feeding practice beyond the second postnatal week. The rapid-progression phenotype was observed only at the Rome site. This is itself an observation about practice: under nutritional protocols that the two units describe as closely aligned, an immediately rapid advancement never occurred at the Brussels site. It also means that contrasts involving that phenotype cannot be separated from center by statistical adjustment, because no Brussels infant belongs to it; they are therefore reported as within-Rome contrasts. More generally, we cannot exclude that the trajectory phenotypes reflect in part the feeding culture of each unit rather than distinct biological subgroups. Whether a trajectory arises from the infant’s tolerance or from the practice of the unit, it is the course the infant actually experienced, and it is that course which is associated with the outcome; a pattern that reflects practice is, if anything, the more modifiable. Both units moreover advanced feeds within the range of the guidelines on which their protocols were based, so the variation described here arose within a codified framework rather than between arbitrary regimens. Cluster membership was estimated from the data and then treated as a known covariate, so the confidence intervals do not incorporate classification uncertainty. Because the clusters were derived from exposure data alone, without reference to outcome, any misclassification would be non-differential and would be expected to attenuate rather than inflate the association, which was in fact larger among infants whose exposure trajectory was completely observed.

Confounding by indication is the principal limitation of this study. Three steps were taken to address it. First, illness severity present before feeding decisions were made was added to the models; the estimate did not fall. Second, morbidities arising after the exposure, together with the proportion of human milk, were entered one at a time; none changed the estimate by more than 10%. Third, an E-value quantified how strong an unmeasured confounder would need to be to explain the association away, though residual confounding cannot be excluded. Socioeconomic status, maternal education, family environment and early intervention services were not recorded in the source datasets; the first two may confound the association, whereas the latter two act between discharge and the 24-month assessment and are therefore sources of uncontrolled variation in the outcome. The cohort is necessarily restricted to infants who survived to the 24-month assessment, so the association described here cannot be extrapolated to infants who die in the neonatal period. Transfer status was not recorded in both source datasets, so infants excluded because of transfer could not be compared with those retained. The two cohorts were assembled from databases covering different calendar periods, the Rome infants having been admitted between 2015 and 2021 and the Brussels infants between 2021 and 2022, so that center and era of care are partially confounded; differences attributed to center may also reflect changes in neonatal practice over time. Infants who did not contribute to the primary outcome had a higher frequency of intraventricular hemorrhage of grade II or above than those analyzed, so the cohort under-represents the most severely affected infants. Such infants would predominantly have belonged to the parenteral-dominant cluster and had poorer outcomes, so their absence is expected to narrow rather than widen the contrast reported here.

## 5. Conclusions

A moderate-progression enteral trajectory during the first two postnatal weeks was associated with better motor neurodevelopment at 24 months: within this cohort the association was confined to a graded progression rather than to either extreme, and it remained statistically significant after adjustment for maturity and did not vary with gestational age. These real-world findings are compatible with divergent evidence within a single non-monotonic dose–response framework and generate the hypothesis, to be tested in a randomized trial enriched for the moderate-risk phenotype and incorporating milk-composition data, that a graded enteral-advancement strategy is a modifiable nutritional target that warrants testing for an effect on long-term motor neurodevelopment. Randomized or prospective multi-center studies incorporating nutritional biomarkers, microbiome analyses and advanced neuroimaging are needed to establish whether the association described here is causal and, if so, at what pace of advancement the benefit is greatest.

## Figures and Tables

**Table 1:** Baseline characteristics of the analytic cohort, overall and by trajectory cluster.

Characteristics	Overall (*n* = 260)	C0 PN-Dominant (*n* = 95)	C1 Moderate (*n* = 116)	C2 Rapid (*n* = 49)	*p*-Value
Center: Rome, n/N (%)	182/260 (70.0%)	73/95 (76.8%)	60/116 (51.7%)	49/49 (100%)	<0.001
IUGR	42/260 (16.2%)	19/95 (20.0%)	13/116 (11.2%)	10/49 (20.4%)	0.150
Antenatal steroids	215/260 (82.7%)	80/95 (84.2%)	94/116 (81.0%)	41/49 (83.7%)	0.815
Multiple pregnancy	80/260 (30.8%)	33/95 (34.7%)	37/116 (31.9%)	10/49 (20.4%)	0.198
Cesarean section	215/260 (82.7%)	79/95 (83.2%)	93/116 (80.2%)	43/49 (87.8%)	0.495
APGAR score at 5 min	8.0 (7.0–9.0)	8.0 (7.0–8.0)	8.0 (7.0–8.2)	8.0 (8.0–9.0)	<0.001
Male sex	131/260 (50.4%)	49/95 (51.6%)	56/116 (48.3%)	26/49 (53.1%)	0.818
Gestational age (weeks)	29.0 (28.0–31.0)	28.0 (26.0–29.0)	30.0 (28.0–31.0)	31.0 (30.0–32.0)	<0.001
Birth-weight z-score	0.1 (−0.6–0.7)	−0.6 (−1.1–0.0)	0.2 (−0.4–0.7)	0.6 (0.3–1.1)	<0.001
Birth-weight percentile	54 (27–74)	28 (14–52)	57 (34–75)	73 (61–86)	<0.001
Head circumference at birth (cm)	27.0 (25.1–28.9)	25.0 (24.0–26.9)	27.5 (26.0–29.0)	28.5 (27.7–29.5)	<0.001

Notes: Data are median (interquartile range) for continuous variables and n/N (%) for categorical variables. Abbreviations: PN, parenteral nutrition; SD, standard deviation.

**Table 2:** Unadjusted Bayley-III composite scores at 24 months of corrected age, by trajectory cluster.

Outcome (24 mo)	Cluster	*n*	Mean (SD)	95% CI	*p*
Motor	C0 PN-dominant	69	90.4 (11.3)	87.6 to 93.1	<0.001
	C1 Moderate	94	98.1 (8.7)	96.4 to 99.9	
	C2 Rapid	31	92.9 (10.5)	89.0 to 96.7	
Cognitive	C0 PN-dominant	62	88.7 (14.0)	85.1 to 92.3	0.068
	C1 Moderate	73	95.8 (13.5)	92.7 to 99.0	
	C2 Rapid	31	93.2 (8.9)	90.0 to 96.5	
Language	C0 PN-dominant	61	80.9 (14.7)	77.2 to 84.7	0.022
	C1 Moderate	71	87.8 (12.5)	84.9 to 90.8	
	C2 Rapid	31	86.9 (9.3)	83.5 to 90.3	

Notes: Data are mean (SD), the 95% confidence interval of the mean, and n per cluster. *p* is the overall comparison across the three clusters (Kruskal–Wallis test). Pairwise comparisons (Mann–Whitney U test) were, for the Motor composite, C0–C1 < 0.001, C0–C2 0.342 and C1–C2 0.020; for the Cognitive composite, 0.022, 0.205 and 0.559; and for the Language composite, 0.009, 0.075 and 0.579. Abbreviations: PN, parenteral nutrition; SD, standard deviation.

**Table 3:** Sensitivity analyses of the primary contrast (moderate-progression versus parenteral-dominant cluster) for the Bayley-III Motor composite at 24 months of corrected age.

Analysis	*n*	Adjusted Difference, Points (95% CI)	*p*
Primary model (random intercept for center)	194	+4.96 (+1.51 to +8.41)	0.005
Center as a fixed effect	194	+4.80 (+1.35 to +8.26)	0.007
Rome center only	128	+2.39 (−2.15 to +6.92)	0.300
Brussels center only	66	+6.71 (+1.39 to +12.03)	0.014
Restricted to birth weight < 1500 g	162	+4.63 (+0.88 to +8.38)	0.016
Restricted to gestational age < 32 weeks	162	+5.10 (+1.23 to +8.96)	0.010
Meeting both eligibility criteria	130	+4.85 (+0.54 to +9.15)	0.027
Complete exposure only, no interpolation	81	+8.24 (+2.20 to +14.27)	0.007
Additional adjustment for baseline severity	194	+5.08 (+1.59 to +8.57)	0.004

Notes: All models are adjusted for gestational age, birth-weight z-score and sex. The primary model includes a random intercept for center; the center-as-fixed-effect model restricts every comparison to infants managed at the same site. Center-stratified estimation and the complete-exposure analysis were pre-specified; the center-as-fixed-effect model, the additional adjustment for baseline severity and the analyses restricted by eligibility criterion were performed post hoc. CI, confidence interval.

## Data Availability

De-identified data are available from the corresponding authors upon reasonable request, subject to approval of the data controllers of both sites.

## Supplementary Materials

The following supporting information can be downloaded at https://www.mdpi.com/article/10.3390/nu18203314/s1. Figure S1, Distribution of the Bayley-III Motor composite at 24 months of corrected age, by trajectory cluster; Figure S2, Effect modification by gestational age of the association between trajectory cluster and the Bayley-III Motor composite at 24 months; Figure S3, Participant flow stratified by center; Table S1, Unadjusted and adjusted between-cluster differences in the three Bayley-III composite scores at 24 months of corrected age; Table S2, Motor delay at 24 months of corrected age, by trajectory cluster; Table S3, Adjusted associations between trajectory cluster and Bayley-III composite scores at 24 months, by adjustment tier; Table S4, Stratification by gestational age of the association between trajectory cluster and the Bayley-III Motor composite at 24 months; Table S5, Nutritional protocol in force at the two participating units during the study period; Table S6, Standardized mean differences for baseline characteristics between trajectory clusters; Table S7, Neonatal morbidities in infants included in and not included in the analytic cohort; Table S8, Neonatal morbidities of the analytic cohort, by trajectory cluster.
