# Supplementary material for: Enteral Feeding Progression Trajectories in the First Two Postnatal Weeks Are Associated with Motor Neurodevelopment at 24 Months in Preterm Infants: A Multicenter Cohort Study
Source: Nutrients. 2026 Oct 9;18(20):3314. doi: 10.3390/nu18203314 (PMC13708045; doi:10.3390/nu18203314)
Supplement: Supplementary file 1 [file nutrients-18-03314-s001.zip › nutrients-4545032-supplementary.pdf]

Supplementary Material

Enteral Feeding Progression Trajectories in the First Two Postnatal Weeks Are Associated with Motor Neurodevelopment at 24 Months in Preterm Infants: A Multicenter Cohort Study

Nutrients — Manuscript ID nutrients-4545032

Contents

Figure S1. Distribution of the Bayley-III Motor composite at 24 months of corrected age, by trajectory cluster.

Figure S2. Effect modification by gestational age of the association between trajectory cluster and the Bayley-III Motor composite at 24 months of corrected age.

Figure S3. Participant flow from the source population to the analytic cohort, stratified by centre.

Table S1. Unadjusted and adjusted between-cluster differences in the three Bayley-III composite scores at 24 months of corrected age.

Table S2. Motor delay (Bayley-III Motor composite below 85) at 24 months of corrected age, by trajectory cluster.

Table S3. Adjusted associations between trajectory cluster and Bayley-III composite scores at 24 months, by adjustment tier. Linear mixed-effects models with a random intercept for centre; all models include gestational age, birth-weight z-score and sex.

Table S4. Stratification by gestational age of the association between trajectory cluster and the Bayley-III Motor composite at 24 months. Panel A reports the formal tests; panel B the stratum-specific estimates, which are exploratory and descriptive.

Table S5. Nutritional protocol in force at the two participating units during the study period.

Table S6. Standardized mean differences for baseline characteristics between trajectory clusters.

Table S7. Neonatal morbidities in infants included in and not included in the analytic cohort.

Table S8. Neonatal morbidities of the analytic cohort, by trajectory cluster.

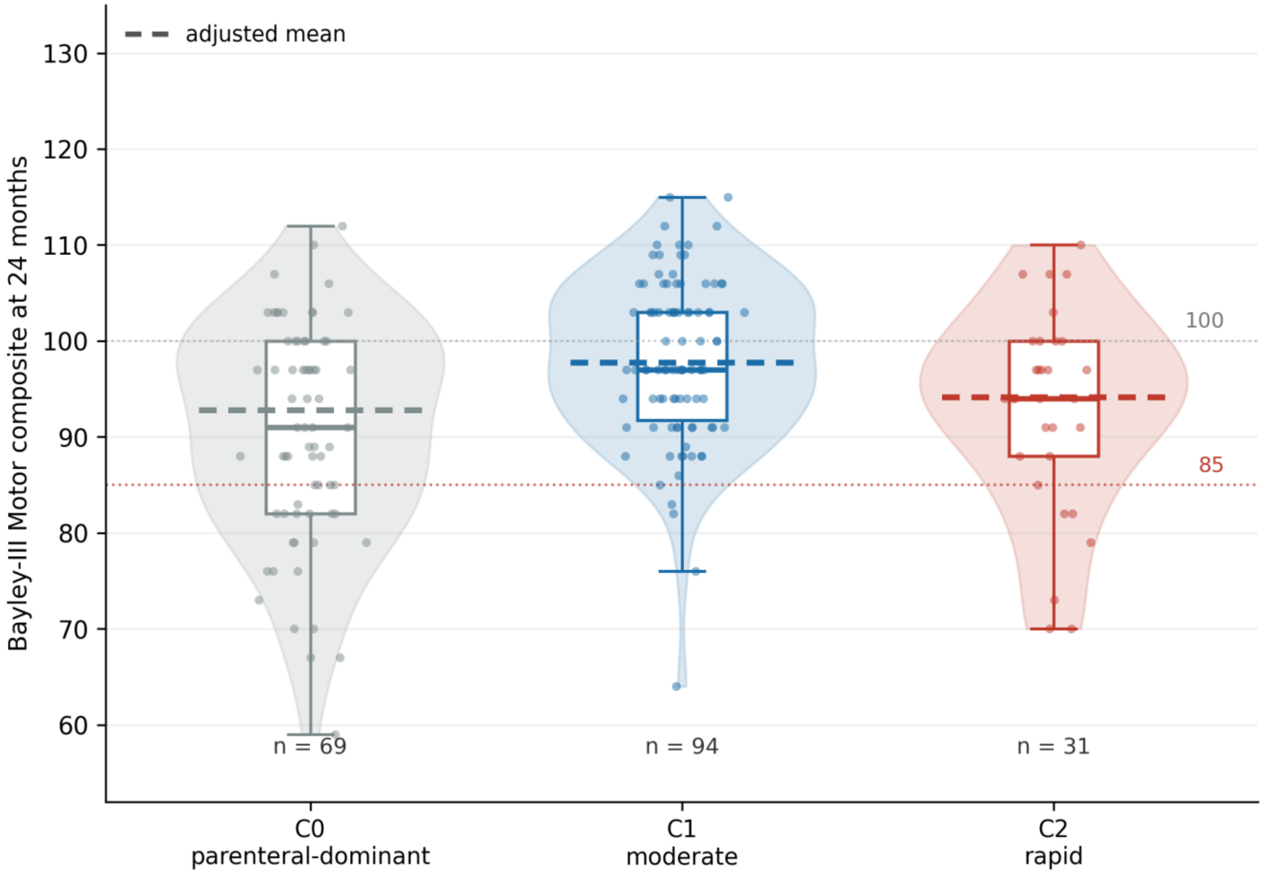

Figure S1. Distribution of the Bayley-III Motor composite at 24 months of corrected age, by trajectory cluster.

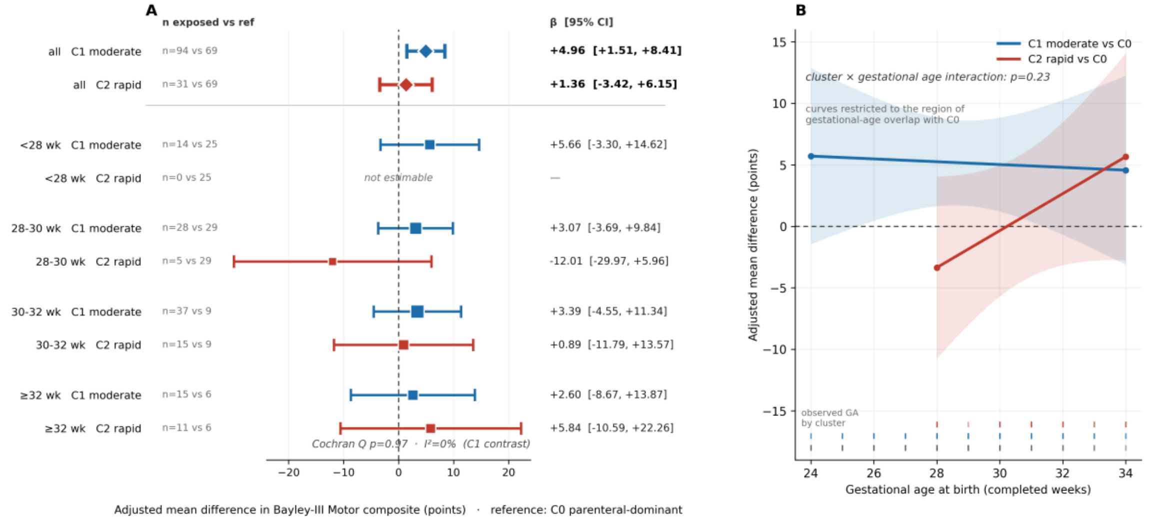

Figure S2. Effect modification by gestational age of the association between trajectory cluster and the Bayley-III Motor composite at 24 months of corrected age.

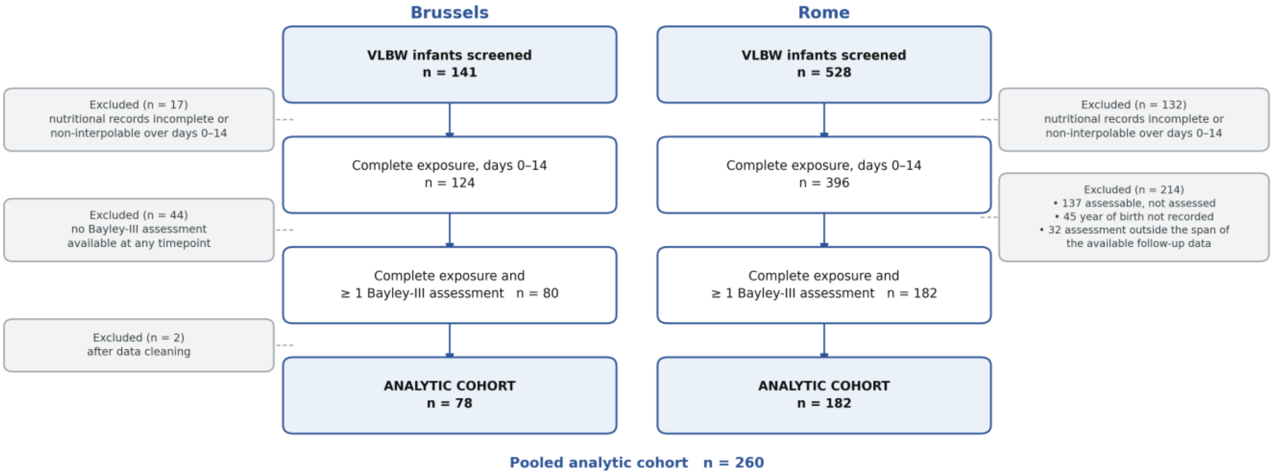

Figure S3. Participant flow by centre. Exclusions are shown beside the column of the unit to which they apply. At the Rome unit, the 214 infants with a complete exposure trajectory but no Bayley-III assessment comprised 137 who were assessable and not assessed, 45 for whom the year of birth was not recorded, and 32 whose 24-month assessment fell outside the span of the follow-up data available for that unit.

Figure S3. Participant flow from the source population to the analytic cohort, stratified by centre.

Table S1. Unadjusted and adjusted between-cluster differences in the three Bayley-III composite scores at 24 months of corrected age.

Domain	Contrast	n	Unadjusted difference	Adjusted β (95% CI)	p	Attenuation
Motor	C1 moderate vs C0	194	+7.78	+4.96 (+1.51, +8.41)	0.005	36%
Motor	C2 rapid vs C0	194	+2.51	+1.36 (-3.42, +6.15)	0.576	46%
Cognitive	C1 moderate vs C0	166	+7.11	+0.17 (-4.20, +4.54)	0.940	98%
Cognitive	C2 rapid vs C0	166	+4.52	+1.73 (-3.99, +7.46)	0.552	62%
Language	C1 moderate vs C0	163	+6.87	+1.80 (-2.91, +6.52)	0.454	74%
Language	C2 rapid vs C0	163	+5.94	+4.58 (-1.52, +10.69)	0.141	23%

Notes: Unadjusted difference: difference in observed cluster means. Adjusted: linear mixed-effects model (cluster + gestational age + birth-weight z-score + sex; random intercept for centre). Attenuation: proportional reduction of the unadjusted difference after adjustment. In both exploratory domains the unadjusted difference favouring the moderate cluster was abolished by adjustment for maturity, whereas the motor difference persisted. Absence of a statistically significant adjusted difference in these two domains is not evidence of absence of an association: the confidence intervals do not exclude differences comparable in magnitude to the motor estimate (cognitive upper bound +4.54 points versus a motor point estimate of +4.96). The Motor composite is the single primary outcome; the Cognitive and Language composites are exploratory and post hoc.

Table S2. Motor delay (Bayley-III Motor composite below 85) at 24 months of corrected age, by trajectory cluster.

Trajectory cluster	n	Motor delay, n (%)	Adjusted odds ratio (95% CI)	p
C0 parenteral-dominant (reference)	69	20 (29.0)	1 [reference]	—
C1 moderate progression	94	4 (4.3)	0.25 (0.07, 0.88)	0.031
C2 rapid progression	31	6 (19.4)	1.52 (0.38, 6.05)	0.552

Notes: Motor delay defined as a Bayley-III Motor composite below 85. Logistic regression adjusted for gestational age, birth-weight z-score, sex and centre (centre as a fixed effect: with only two centres a random-effect variance is not identifiable). Events are few — four in the moderate cluster — so the estimate is imprecise and the confidence interval wide. Severe motor delay (composite below 70) occurred in four infants overall and was not analysed inferentially. Pre-specified secondary outcome.

Table S3. Adjusted associations between trajectory cluster and Bayley-III composite scores at 24 months, by adjustment tier. Linear mixed-effects models with a random intercept for centre; all models include gestational age, birth-weight z-score and sex.

Model	Domain	Contrast	n	β	95% CI	*p*
Tier A — primary model	Motor	C1 moderate vs C0	194	4.96	+1.51 to +8.41	0.005
	Motor	C2 rapid vs C0	194	1.36	-3.42 to +6.15	0.576
	Motor	C2 rapid vs C1	194	-3.59	-7.93 to +0.74	0.104
	Cognitive	C1 moderate vs C0	166	0.17	-4.20 to +4.54	0.940
	Cognitive	C2 rapid vs C0	166	1.73	-3.99 to +7.46	0.552
	Cognitive	C2 rapid vs C1	166	1.57	-3.40 to +6.54	0.537
	Language	C1 moderate vs C0	163	1.80	-2.91 to +6.52	0.454
	Language	C2 rapid vs C0	163	4.58	-1.52 to +10.69	0.141
	Language	C2 rapid vs C1	163	2.78	-2.49 to +8.05	0.302
Tier B — + 5-min Apgar + surfactant + antenatal corticosteroids	Motor	C1 moderate vs C0	194	5.08	+1.59 to +8.57	0.004
	Motor	C2 rapid vs C0	194	1.75	-3.20 to +6.70	0.488
	Motor	C2 rapid vs C1	194	-3.33	-7.69 to +1.03	0.135
	Cognitive	C1 moderate vs C0	166	0.48	-3.98 to +4.94	0.833
	Cognitive	C2 rapid vs C0	166	2.07	-3.92 to +8.06	0.498
	Cognitive	C2 rapid vs C1	166	1.59	-3.46 to +6.64	0.538
	Language	C1 moderate vs C0	163	2.69	-2.09 to +7.47	0.270
	Language	C2 rapid vs C0	163	6.48	+0.14 to +12.82	0.045
	Language	C2 rapid vs C1	163	3.79	-1.53 to +9.11	0.163
Tier C — + mechanical ventilation	Motor	C1 moderate vs C0	187	4.46	+0.90 to +8.02	0.014
	Motor	C2 rapid vs C0	187	-0.28	-5.54 to +4.99	0.918
	Motor	C2 rapid vs C1	187	-4.74	-9.40 to -0.07	0.047
	Cognitive	C1 moderate vs C0	159	0.19	-4.38 to +4.77	0.934
	Cognitive	C2 rapid vs C0	159	0.89	-5.40 to +7.19	0.781
	Cognitive	C2 rapid vs C1	159	0.70	-4.59 to +5.99	0.795
	Language	C1 moderate vs C0	156	1.67	-3.16 to +6.50	0.498
	Language	C2 rapid vs C0	156	2.55	-4.02 to +9.13	0.447
	Language	C2 rapid vs C1	156	0.88	-4.59 to +6.36	0.752
Tier C — + bronchopulmonary dysplasia	Motor	C1 moderate vs C0	189	5.16	+1.60 to +8.73	0.005
	Motor	C2 rapid vs C0	189	1.20	-3.63 to +6.04	0.626
	Motor	C2 rapid vs C1	189	-3.96	-8.36 to +0.44	0.078
	Cognitive	C1 moderate vs C0	165	-0.30	-4.90 to +4.30	0.898
	Cognitive	C2 rapid vs C0	165	1.65	-4.12 to +7.43	0.575
	Cognitive	C2 rapid vs C1	165	1.95	-3.10 to +7.01	0.449
	Language	C1 moderate vs	162	1.64	-3.33 to +6.61	0.518

		C0				
	Language	C2 rapid vs C0	162	4.72	-1.43 to +10.88	0.133
	Language	C2 rapid vs C1	162	3.08	-2.27 to +8.44	0.259
Tier C — + necrotising enterocolitis	Motor	C1 moderate vs C0	194	4.69	+1.21 to +8.18	0.008
	Motor	C2 rapid vs C0	194	1.25	-3.53 to +6.04	0.607
	Motor	C2 rapid vs C1	194	-3.44	-7.78 to +0.91	0.121
	Cognitive	C1 moderate vs C0	166	0.20	-4.24 to +4.65	0.929
	Cognitive	C2 rapid vs C0	166	1.75	-4.00 to +7.50	0.551
	Cognitive	C2 rapid vs C1	166	1.55	-3.46 to +6.55	0.545
	Language	C1 moderate vs C0	163	1.52	-3.27 to +6.32	0.534
	Language	C2 rapid vs C0	163	4.45	-1.67 to +10.58	0.154
	Language	C2 rapid vs C1	163	2.93	-2.37 to +8.23	0.278
Tier C — + intraventricular haemorrhage	Motor	C1 moderate vs C0	194	4.90	+1.46 to +8.35	0.005
	Motor	C2 rapid vs C0	194	1.33	-3.44 to +6.11	0.584
	Motor	C2 rapid vs C1	194	-3.57	-7.90 to +0.76	0.106
	Cognitive	C1 moderate vs C0	166	-0.00	-4.39 to +4.38	0.998
	Cognitive	C2 rapid vs C0	166	1.61	-4.11 to +7.34	0.581
	Cognitive	C2 rapid vs C1	166	1.62	-3.35 to +6.59	0.524
	Language	C1 moderate vs C0	163	1.49	-3.22 to +6.19	0.536
	Language	C2 rapid vs C0	163	4.35	-1.72 to +10.43	0.160
	Language	C2 rapid vs C1	163	2.87	-2.37 to +8.11	0.284
Tier C — + late-onset sepsis	Motor	C1 moderate vs C0	194	4.96	+1.28 to +8.64	0.008
	Motor	C2 rapid vs C0	194	1.36	-3.48 to +6.20	0.582
	Motor	C2 rapid vs C1	194	-3.60	-7.99 to +0.79	0.108
	Cognitive	C1 moderate vs C0	166	-0.50	-4.99 to +3.99	0.828
	Cognitive	C2 rapid vs C0	166	1.44	-4.29 to +7.17	0.621
	Cognitive	C2 rapid vs C1	166	1.94	-3.05 to +6.94	0.446
	Language	C1 moderate vs C0	163	1.38	-3.50 to +6.26	0.579
	Language	C2 rapid vs C0	163	4.39	-1.75 to +10.53	0.161
	Language	C2 rapid vs C1	163	3.01	-2.31 to +8.33	0.268
Tier C — + postnatal corticosteroids	Motor	C1 moderate vs C0	191	4.70	+1.16 to +8.25	0.009
	Motor	C2 rapid vs C0	191	1.19	-3.62 to +5.99	0.628
	Motor	C2 rapid vs C1	191	-3.52	-7.88 to +0.85	0.114
	Cognitive	C1 moderate vs C0	165	0.01	-4.49 to +4.51	0.996
	Cognitive	C2 rapid vs C0	165	1.66	-4.12 to +7.45	0.573
	Cognitive	C2 rapid vs C1	165	1.65	-3.35 to +6.65	0.517
	Language	C1 moderate vs C0	162	2.03	-2.83 to +6.89	0.413
	Language	C2 rapid vs C0	162	4.79	-1.38 to +10.96	0.128
	Language	C2 rapid vs C1	162	2.76	-2.53 to +8.06	0.306

Tier C — + human-milk fraction	Motor	C1 moderate vs C0	189	4.64	+1.11 to +8.17	0.010
	Motor	C2 rapid vs C0	189	0.93	-3.95 to +5.81	0.710
	Motor	C2 rapid vs C1	189	-3.72	-8.11 to +0.67	0.097
	Cognitive	C1 moderate vs C0	161	-0.85	-5.25 to +3.54	0.704
	Cognitive	C2 rapid vs C0	161	0.44	-5.30 to +6.19	0.880
	Cognitive	C2 rapid vs C1	161	1.29	-3.62 to +6.21	0.606
	Language	C1 moderate vs C0	158	1.09	-3.68 to +5.86	0.654
	Language	C2 rapid vs C0	158	3.48	-2.68 to +9.64	0.268
	Language	C2 rapid vs C1	158	2.39	-2.86 to +7.64	0.373

Notes: β denotes the adjusted mean difference in composite score (points). C0, parenteral-dominant; C1, moderate progression; C2, rapid progression. Rows shown in red are new or revised in this revision. The Motor composite at 24 months is the single pre-specified primary outcome; the Cognitive and Language domains are exploratory.

Table S4. Stratification by gestational age of the association between trajectory cluster and the Bayley-III Motor composite at 24 months. Panel A reports the formal tests; panel B the stratum-specific estimates, which are exploratory and descriptive.

Test	Statistic	p	Interpretation
Cluster × gestational age interaction (gestational age continuous)	LRT χ^2=2.91, df=2	0.23	no evidence of effect modification
Cluster × gestational-age stratum interaction (four strata)	LRT χ^2=3.96, df=6	0.68	confirmatory, less powerful
Cluster × gestational age interaction within the Rome centre	LRT χ^2=2.57, df=2	0.28	confirmed with centre held fixed
Heterogeneity of the C1-versus-C0 estimate across strata	Cochran Q=0.26, df=3, I^2=0%	0.97	stratum estimates homogeneous
Sensitivity: gestational age with a quadratic term	C1 vs C0 β=+5.06 (linear +4.96)	0.004	no residual functional-form confounding
Sensitivity: restricted to gestational age <32 weeks (n=162)	C1 vs C0 β=+5.10	0.010	unchanged excluding infants eligible by birth weight alone

**Gestational age**	**Contrast**	**n exposed vs reference**	**Adjusted β (95% CI)**	**p**	**Power for a 5-point effect**
Whole cohort	C1 moderate vs C0	94 vs 69	+4.96 (+1.51, +8.41)	0.005	—
<28 weeks	C1 moderate vs C0	14 vs 25	+5.66 (−3.30, +14.62)	0.22	32%
28–30 weeks	C1 moderate vs C0	28 vs 29	+3.07 (−3.69, +9.84)	0.37	47%
30–32 weeks	C1 moderate vs C0	37 vs 9	+3.39 (−4.55, +11.34)	0.40	27%
≥32 weeks	C1 moderate vs C0	15 vs 6	+2.60 (−8.67, +13.87)	0.65	18%
Whole cohort	C2 rapid vs C0	31 vs 69	+1.36 (−3.42, +6.15)	0.58	—
<28 weeks	C2 rapid vs C0	0 vs 25	not estimable	—	—
28–30 weeks	C2 rapid vs C0	5 vs 29	−12.01 (−29.97, +5.96)	0.19	—
30–32 weeks	C2 rapid vs C0	15 vs 9	+0.89 (−11.79, +13.57)	0.89	—
≥32 weeks	C2 rapid vs C0	11 vs 6	+5.84 (−10.59, +22.26)	0.49	—

A. Formal tests

B. Stratum-specific estimates

Notes: Stratum-specific estimates were obtained by ordinary least squares with HC3 robust standard errors, adjusted for gestational age, birth-weight z-score, sex and centre. Contrasts are reported as non-estimable where a cluster contained fewer than five infants within a stratum, rather than estimated by model extrapolation. The fourth stratum comprises infants eligible by birth weight alone. Within strata, cluster membership becomes closely entangled with centre: fully within-centre stratified estimates were largely non-estimable. These analyses were post hoc.

Table S5. Nutritional protocol in force at the two participating units during the study period.

Parameter	Rome — birth weight <1000 g	Rome — birth weight ≥1000 g	Brussels
**PARENTERAL NUTRITION**			
Initiation	Within 24 h of life	Within 24 h of life	Within 24 h of life
Access	Central venous or PICC	Central venous or PICC	Central venous or PICC
Prescription	Individualised daily on clinical condition, laboratory results and weight	Individualised daily on clinical condition, laboratory results and weight	Ready to use 3 chamber PN
Energy, starting dose	45–58 kcal/kg/day	40–45 kcal/kg/day	40–45 kcal/kg/day
Energy, target dose	100–110 kcal/kg/day	90–100 kcal/kg/day	90–100 kcal/kg/day
Energy, days to target	4–5	4–5	4–5
Amino acids, starting dose	2.0–2.5 g/kg/day	3.2–3.5 g/kg/day	2–3.5 g/kg/day
Amino acids, target dose	3.5–4.0 g/kg/day	3.5–4.0 g/kg/day	3.5–4.0 g/kg/day
Amino acids, days to target	3–4	3–4	3–4
Dextrose, starting dose	6.5–7.0 g/kg/day	6.5–7.0 g/kg/day	6.5–7.0 g/kg/day
Dextrose, target dose	13.0–14.0 g/kg/day	13.0–14.0 g/kg/day	13.0–14.0 g/kg/day
Dextrose, days to target	5–7	5–7	5–7
Lipid emulsion	Intralipid	Intralipid	ClinOleic
Lipids, starting dose	1.5–2.0 g/kg/day	1.0–1.5 g/kg/day	1.5–2.0 g/kg/day
Lipids, target dose	3.5–4.0 g/kg/day	3.0–3.5 g/kg/day	3.5–4.0 g/kg/day
Lipids, days to target	3–5	3–5	3–4
Calcium (calcium gluconate 10%)	40 → 80 mg/kg/day by day 8	40 → 80 mg/kg/day by day 8	40 → 80 mg/kg/day by day 8
Phosphorus (sodium glycerophosphate)	25 → 75 mg/kg/day by day 8; P/Ca ratio 1	25 → 75 mg/kg/day by day 8; P/Ca ratio 1	25 → 75 mg/kg/day by day 8; P/Ca ratio 1
**TOTAL FLUID INTAKE (PN + EN)**			
Starting volume	70–90 mL/kg/day	70–90 mL/kg/day	70–90 mL/kg/day
Daily increment	10–20 mL/kg/day	10–20 mL/kg/day	10–20 mL/kg/day
Target volume	150–180 mL/kg/day	150–180 mL/kg/day	150–180 mL/kg/day
**ENTERAL NUTRITION**			
Initiation	Within 96 h of life, once clinically stable	Within 96 h of life, once clinically stable	Within 96 h of life, once clinically stable
Minimal enteral feeding	10–20 mL/kg/day in 4–8 feeds	10–20 mL/kg/day in 4–8 feeds	10–20 mL/kg/day in 8 feeds
Daily advancement	15–30 mL/kg/day by birth weight and tolerance	15–30 mL/kg/day by birth weight and tolerance	15–30 mL/kg/day by tolerance
Milk at initiation	Mother's own milk, fresh when available	Mother's own milk, fresh when available	Mother's own milk, fresh when available
Donor human milk	Not available	Not available	Not available
Fortification threshold	Enteral volume 100 mL/kg/day	Enteral volume 80–100 mL/kg/day	Enteral volume 80 mL/kg/day
Fortifier	FM 85 (Nestlé)	FM 85 (Nestlé)	Pre NAN HMF (Nestlé)
Probiotics	Not administered	Not administered	Administered
Full enteral feeding	120 kcal/kg/day	120 kcal/kg/day	120 kcal/kg/day
Feeding intolerance	Significant abdominal distension, emesis, ileus with visible intestinal loops, blood in the stools; or haemodynamic instability	Same	Same
Withholding	≥ 6 h, then re-evaluation before resumption	≥ 6 h, then re-evaluation before resumption	≥ 6 h, then re-evaluation before resumption

Notes: The protocol shown is the one in force at each unit during the study period. Parenteral nutrition was prescribed individually each day within the ranges given at the Rome unit and delivered as a ready-to-use three-chamber bag at the Brussels unit. PICC, peripherally inserted central catheter; HMF, human-milk fortifier.

Table S6. Standardized mean differences for baseline characteristics between trajectory clusters.

Characteristic	n	C0 vs C1	C0 vs C2	C1 vs C2
Center: Rome	260	+0.54	-0.78	-1.37
IUGR	260	+0.24	-0.01	-0.25
Antenatal steroids	260	+0.08	+0.01	-0.07
Multiple pregnancy	260	+0.06	+0.32	+0.26
Cesarean section	260	+0.08	-0.13	-0.21
APGAR score at 5 min	260	-0.30	-0.78	-0.53
Male sex	260	+0.07	-0.03	-0.10
Gestational age (weeks)	260	-0.73	-1.45	-0.76
Birth weight z-score	260	-0.93	-1.76	-0.79
Head circumference at birth (cm)	259	-0.83	-1.49	-0.61

Notes: Standardized mean differences are given for each pairwise comparison; values are computed from means and standard deviations for continuous variables and from proportions for binary variables. An absolute value above 0.10 is conventionally taken to indicate imbalance. C0, parenteral-dominant; C1, moderate progression; C2, rapid progression.

Table S7. Neonatal morbidities in infants included in and not included in the analytic cohort.

Characteristic	Included (n = 260)	Not included (n = 409)	*p*
Bronchopulmonary dysplasia at 36 weeks	29/255 (11.4%)	35/391 (9.0%)	0.383
Intraventricular haemorrhage, grade ≥II	15/260 (5.8%)	49/405 (12.1%)	0.010
Necrotising enterocolitis, Bell stage ≥II	13/260 (5.0%)	24/330 (7.3%)	0.337
Late-onset sepsis	34/258 (13.2%)	66/401 (16.5%)	0.301
Mechanical ventilation	148/244 (60.7%)	118/229 (51.5%)	0.057

Notes: data are n/N (%) and were compared with the chi-square test; denominators give the number of infants with the variable recorded. Late-onset sepsis was defined as clinical or culture-proven sepsis with onset after the seventh postnatal day at the Rome unit; at the Brussels unit the variable recorded by the source database was used. * $p < 0.05$.

Table S8. Neonatal morbidities of the analytic cohort, by trajectory cluster.

Morbidity	C0 parenteral-dominant	C1 moderate	C2 rapid	*p*
Mechanical ventilation (any, first week)	72/87 (82.8%)	51/114 (44.7%)	25/43 (58.1%)	<0.001
Bronchopulmonary dysplasia (36 weeks)	22/94 (23.4%)	7/112 (6.2%)	0/49 (0.0%)	<0.001
Necrotising enterocolitis (Bell stage ≥ 2)	7/95 (7.4%)	4/116 (3.4%)	2/49 (4.1%)	0.407
Intraventricular haemorrhage (grade ≥ 2)	9/95 (9.5%)	6/116 (5.2%)	0/49 (0.0%)	0.065
Late-onset sepsis (clinical or culture-proven)	28/95 (29.5%)	9/116 (7.8%)	1/49 (2.0%)	<0.001
Retinopathy of prematurity (any stage)	38/95 (40.0%)	14/110 (12.7%)	4/49 (8.2%)	<0.001

Notes: Data are n/N (%); denominators give the number of infants in whom the variable was recorded. These morbidities occur during or after the exposure window of postnatal days 0–14 and are therefore not baseline characteristics; they are reported to describe the clinical profile of the cohort and were not entered in the primary model. Retinopathy of prematurity was recorded as presence of any stage at the Rome unit and as stage 1 or 2 at the Brussels unit. Groups were compared with the chi-square test.
